# Role of weight-adjusted waist circumference index and non-high-density lipoprotein cholesterol/high-density lipoprotein cholesterol ratio in prediabetes risk: A mediation analysis

**DOI:** 10.1371/journal.pone.0331866

**Published:** 2025-09-25

**Authors:** Wei Mi, Cuixiao Wang

**Affiliations:** 1 School of Nursing, Hunan University of Medicine, Changsha, China; 2 Hunan University of Medicine General Hospital, Changsha, Hunan, China; University of Montenegro-Faculty of Medicine, MONTENEGRO

## Abstract

**Objective:**

This study aims to investigate the prevalence of prediabetes among U.S. adults using NHANES 2017–2023 data, explore the relationship between the weight-adjusted waist circumference index (WWI) and the non-high-density lipoprotein cholesterol/high-density lipoprotein cholesterol ratio (NHHR) and the risk of prediabetes, and provide a theoretical foundation for the early identification and intervention of prediabetes.

**Methods:**

Logistic regression, linear regression model, subgroup analysis, restricted cubic spline (RCS) analysis, and mediation analysis were employed to examine the effect of WWI on prediabetes and the mediating role of NHHR.

**Results:**

A total of 9,713 adults were included in the analysis, with an age range of 20–80 and a mean age of 51.26 ± 17.25. Of these, 3,208 cases (33.03%) were prediabetic. The adjusted model showed that WWI (OR = 1.55, 95% CI: 1.46, 1.64) may increase the probability of prediabetes. The results of subgroup analysis, stratified by factors such as gender and age, largely supported these findings. The adjusted model also indicated that NHHR (OR = 1.20, 95% CI: 1.16, 1.25) may increase the probability of prediabetes. The RCS analysis revealed a nonlinear relationship between both WWI and NHHR with the risk of prediabetes. Further mediation analysis indicated that NHHR mediated 10% of the effect of WWI on prediabetes.

**Conclusion:**

WWI and NHHR were significantly associated with the risk of prediabetes, and NHHR partially mediated the effect of WWI on prediabetes. This study provides a new theoretical basis for early prediabetes screening, prevention, and control. In the future, interventions targeting WWI and NHHR through lifestyle changes may be effective in preventing prediabetes.

## 1. Introduction

As early as 2000, diabetes was positioned within the top 10 causes of death worldwide, and its prevalence is projected to reach 10.2% (578 million) of the global population by 2030 [1], with direct health expenditures for diabetes expected to grow to $825 billion [2]. Diabetes increases mortality risk and contributes to multiple chronic complications that impose a significant burden on patients’ lives [3]. Before diagnosis, individuals with type 2 diabetes almost always go through a prediabetic phase characterized by elevated blood glucose levels, defined as impaired fasting glucose (IFG) or impaired glucose tolerance (IGT), but not sufficient to meet the criteria for diabetes [4].

Prediabetes is not only a contributing factor for type 2 diabetes. However, it is also closely associated with a variety of other diseases, and patients with prediabetes face a higher risk of cardiovascular disease (CVD) [5], metabolic syndrome [6], and other diseases. Some studies have shown that prediabetes can elevate all-cause mortality in patients with atherosclerotic cardiovascular disease [7]. Moreover, prediabetes is strongly linked to an elevated risk of developing various cancers [8].

According to the World Health Organization, the global prevalence of prediabetes is rapidly increasing, and if left unaddressed, more than 470 million people will have prediabetes by 2030 [9]. As prediabetes often have no obvious symptoms, many individuals are unaware that they are at high risk. In the absence of effective interventions, prediabetes frequently progresses to type 2 diabetes at an annual conversion rate between 5% and 10% [9]. Therefore, early screening and intervention for prediabetes are critical.

The development of prediabetes is closely associated with several factors. Metabolic factors such as insulin resistance, obesity, and dyslipidemia significantly increase the risk of prediabetes [4]. Dyslipidemia plays a significant role in the progression of diabetes mellitus. Elevated levels of non-high-density lipoprotein cholesterol (non-HDL-C), including low-density lipoprotein cholesterol (LDL-C) and very low-density lipoprotein remnants, primarily characterize this condition [10].In recent years, NHHR has gained widespread attention. Compared with non-HDL-C, NHHR is a comprehensive and sensitive index that considers both atherosclerosis risk factors (non-HDL-C) and beneficial factors (HDL-C), making it effective in predicting all-cause mortality, as well as cardiovascular mortality, in patients with diabetes mellitus or prediabetes [11].On the other hand, the weight-adjusted waist index (WWI) is a new central obesity assessment metric, an anthropometric index introduced in 2018 [12].WWI combines the advantages of waist circumference and overcomes the limitations of BMI, offering a more precise evaluation of adiposity and muscle tissue. It has been widely utilized in clinical studies of cardiovascular disease [13], sarcopenia [14], and nephropathy [15].

We hypothesized that higher WWI and NHHR are linked to an elevated risk of prediabetes and that WWI influences the onset of prediabetes through NHHR. Therefore, this study aimed to assess the prevalence of prediabetes and the association between WWI and NHHR with the risk of prediabetes in U.S. adults, using data from the NHANES 2017–2023 survey to provide a theoretical basis for the early identification and intervention of prediabetes.

## 2. Materials and methods

### 2.1. Data sources and sample selection

This study used data from the NHANES 2017–2023 survey, which included 27,763 participants. These data were ethically approved by the NHANES Medical Ethics Committee, with all participants providing signed informed consent, obviating the need for additional ethical approval. Participants with missing data on variables such as race, age, educational status, and smoking were excluded, and 9,713 adults were ultimately included in the analysis, as shown in S1 Fig.

### 2.2. Definition of prediabetes

(1) HbA1c: between 5.7% and 6.4%, (2) FBG: between 5.6 and 6.9 mmol/L, and (3) 2hPG: ranging from 7.8 to 11.0 mmol/L [11].

### 2.3. Definition of WWI

The measurements of the subjects were performed by trained medical personnel, ensuring the accuracy of the measurements to the greatest extent possible. WWI was derived by dividing waist circumference (cm) by the square root of body weight (kg) [12].

### 2.4. NHHR definitions

The official NHANES website provides detailed information on collecting, preserving, and transporting blood samples, as well as testing laboratory indicators. The calculation formula is as follows [16]:


Non−HDL−C=Total Cholesterol (TC)−HDL−C

NHHR=Non−HDL−C/HDL−C

### 2.5. Covariates

The covariates controlled in this study included race, sex, age, poverty-income ratio (PIR), education level, smoking status, marital status, drinking status, hypertension, and coronary heart disease. These covariates were obtained through measurements or standardized questionnaires. Medical history was defined as present or absent based on the subjects’ self-reported status.

### 2.6. Statistical methods

Data from this study were organized and, analyzed statistically with R software. Continuous variables are presented as means ± standard deviation, and comparisons were made using independent samples t-tests. Categorical variables are reported as frequencies and percentages, with the χ² test used for one-way analyses of variance. Influence factor analysis was conducted using logistic regression models and linear regression models; two models were constructed: the crude model without adjusting for variables and the adjusted model with all covariates included. Subgroup analyses were performed to validate WWI’s effect on prediabetes further, and a restricted cubic spline (RCS) analysis was used to assess the impact of WWI and NHHR on prediabetes. A bilateral P-value of less than 0.05 was regarded as statistically significant.

## 3. Results

### 3.1. Baseline characteristics

A total of 9,713 adults were included in the analysis, with an age range of 20–80 and a mean age of 51.26 ± 17.25. Of these, 3,208 cases (33.03%) were prediabetic. The mean WWI was 11.09 ± 0.84, and the mean NHHR was 2.67 ± 1.22. Except for gender and marital status, the prevalence of prediabetes differed significantly across all other groups. The characteristics of the participants are presented in Table 1.

**Table 1:** Baseline Characteristics of Study Participants.

Characteristics	Total (n = 9,713)	Non-prediabetes (n = 6,505)	Prediabetes (n = 3,208)	*P* Value
Age (n (%))				
20–65	7158 (73.7)	5148 (79.1)	2010 (62.7)	<0.001
65–80	2555 (26.3)	1357 (20.9)	1198 (37.3)	
Race (n (%))				
White	4695 (48.3)	3365 (51.7)	1330 (41.5)	<0.001
Black and others	5018 (51.7)	3140 (48.3)	1878 (58.5)	
Sex (n (%))				
male	4636(47.7)	3070 (47.2)	1566 (48.8)	0.25
female	5077(52.3)	3435 (52.8)	1642 (51.2)	
Marital status (n (%))				
Single/divorced/widowed/ separated	5602 (57.7)	3758 (57.8)	1844 (57.5)	0.803
Married/cohabited	4111 (42.3)	2747 (42.2)	1364 (42.5)	
Education level (n (%))				
Below high school	1319 (13.6)	793 (12.2)	526 (16.4)	<0.001
High school	2127 (21.9)	1378 (21.2)	749 (23.3)	
Above high school	6267 (64.5)	4334 (66.6)	1933 (60.3)	
PIR(n(%))				
<2	3769 (38.8)	2454 (37.7)	1315 (41.0)	0.002
≥2	5944 (61.2)	4051 (62.3)	1893 (59.0)	
CHD (n (%))				
No	9297 (95.7)	6304 (96.9)	2993 (93.3)	<0.001
Yes	416 (4.3)	201 (3.1)	215 (6.7)	
Stroke (n (%))				
No	9316 (95.9)	6288 (96.7)	3028 (94.4)	<0.001
Yes	397 (4.1)	217 (3.3)	180 (5.6)	
Hypertension (n (%))				
No	6262 (64.5)	4561 (70.1)	1701 (53.0)	<0.001
Yes	3451 (35.5)	1944 (29.9)	1507 (47.0)	
Drinking status (n (%))				
No	781 (8.0)	474 (7.3)	307 (9.6)	<0.001
Yes	8932 (92.0)	6031 (92.7)	2901 (90.4)	
Smoking status (n (%))				
No	5661 (58.3)	3887 (59.8)	1774 (55.3)	<0.001
Yes	4052 (41.7)	2618 (40.2)	1434 (44.7)	
NHHR (mean (SD))	2.67 (1.22)	2.60 (1.22)	2.81 (1.21)	<0.001
WWI (mean (SD))	11.09 (0.84)	10.96 (0.85)	11.34 (0.77)	<0.001

### 3.2. Effect of WWI on prediabetes

**Fig 1:**
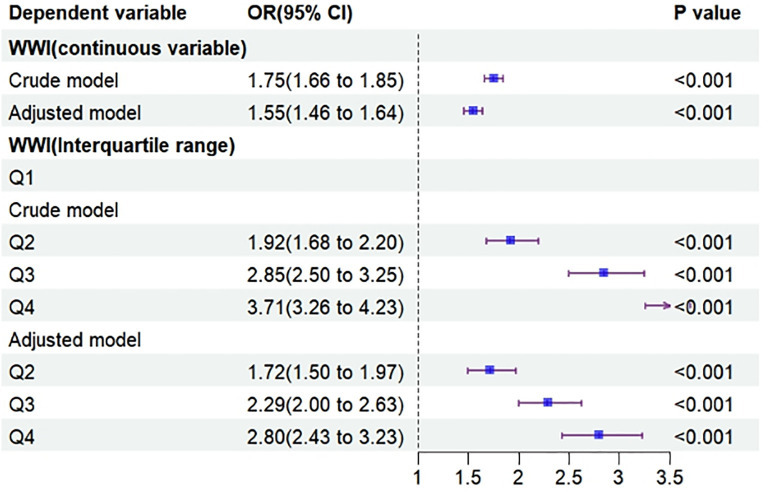
Forest Plot of the Effect of WWI on Prediabetes.

Logistic regression analysis of WWI as a continuous variable with prediabetes as the dependent variable showed that both the crude model’s (OR = 1.75, 95% CI: 1.66, 1.85) and the adjusted model’s (OR = 1.55, 95% CI: 1.46, 1.64) results indicated that WWI may increase the probability of prediabetes occurrence (see Fig 1). When WWI was analyzed in logistic regression with prediabetes as a categorical variable in quartiles, the likelihood of developing prediabetes increased with higher WWI compared to Q1, further supporting the above results (see Fig 1).

**Fig 2:**
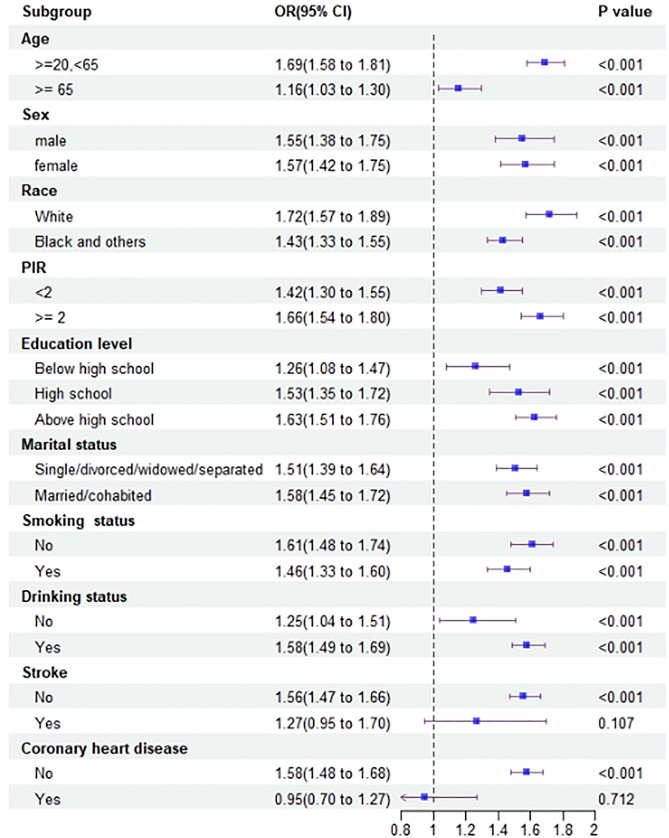
Forest plot of subgroup analysis of the effect of WWI on prediabetes.

**Fig 3:**
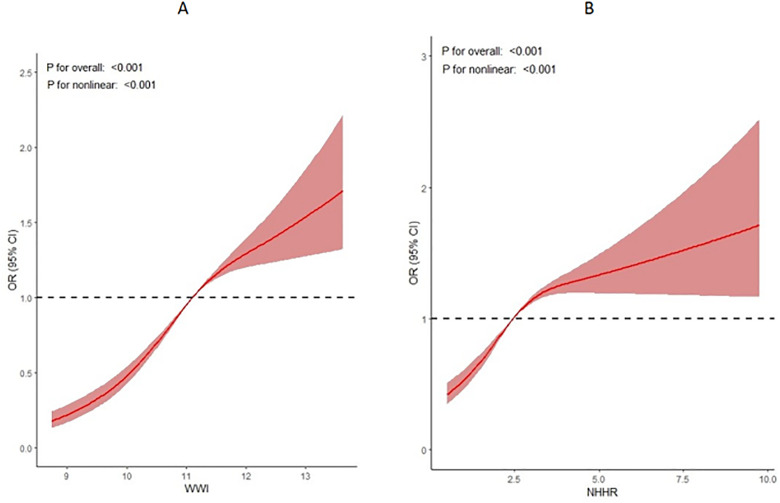
RCS results: A, WWI and the risk of developing prediabetes; B, NHHR and the risk of developing prediabetes].

The subgroups were categorized based on all covariates. Logistic regression analysis was performed within each group, with WWI as the independent variable and prediabetes as the dependent variable. Except for the heterogeneity observed in the stroke and coronary heart disease groups, the results of the other subgroup analyses were consistent with the above findings (see Fig 2). The outcomes of the RCS analysis revealed a nonlinear relationship between WWI and the risk of prediabetes, as shown in Fig 3A.

### 3.3. Effect of NHHR on prediabetes

**Fig 4:**
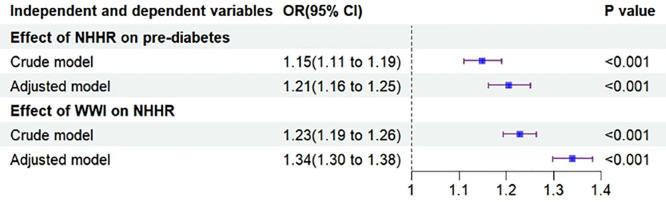
Forest plot illustrating the associations between NHHR and prediabetes, as well as between WWI and NHHR.

Logistic regression analysis with NHHR as the independent variable and prediabetes as the dependent variable showed that NHHR may increase the probability of prediabetes, both in the crude model (OR = 1.15, 95% CI: 1.11, 1.19) and in the adjusted model (OR = 1.21, 95% CI: 1.16, 1.25), as shown in Fig 4. The results of the RCS analysis revealed a nonlinear relationship between NHHR and the risk of prediabetes (see Fig 3B).

### 3.4. Effect of WWI on NHHR

Multiple linear regression analysis was performed with WWI as the independent variable and NHHR as the dependent variable. The effect of WWI on NHHR was significant in both the crude model (OR = 1.23, 95% CI: 1.19, 1.26) and the adjusted model (OR = 1.34, 95% CI: 1.30, 1.38), as shown in Fig 4.

### 3.5. Results of mediation analysis

**Fig 5:**
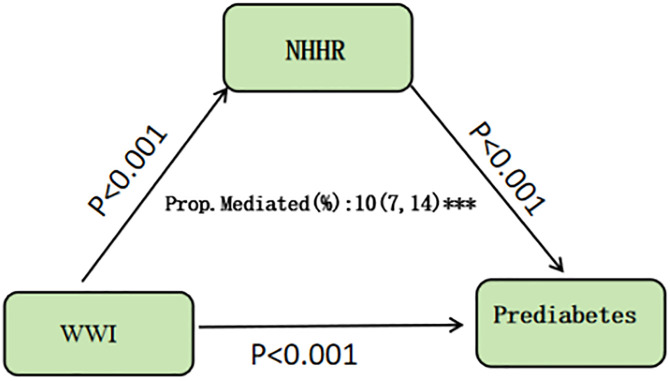
Results of mediation analysis.

Mediation analysis was performed using the bootstrap method, with WWI as the independent variable, prediabetes as the dependent variable, and NHHR as the mediator variable, using 5,000 replicate samples. The results showed that NHHR mediated 10% of the effect of WWI on prediabetes (see Fig 5).

## 4. Discussion

This study, involving 9,713 U.S. adults, explored the relationship between WWI and the NHHR with the risk of prediabetes. Studies have shown that the prevalence of prediabetes among U.S. adults aged 20–80 years from 2017–2023 is 33.03%, slightly higher than the prevalence of prediabetes among adolescents in Sudan (32.6%) [17]. In our study, WWI and NHHR were significantly correlated with the risk of prediabetes, and further analyses revealed that NHHR partially mediated the effect of WWI on prediabetes.

This study demonstrated that WWI is strongly associated with the risk of prediabetes, with each one-unit increase in WWI corresponding to a 0.55-fold increase in risk. WWI has been shown to significantly correlate with the development of diabetes mellitus and complications such as diabetic retinopathy [18] and diabetic nephropathy [15].WWI combines waist circumference and height as a comprehensive index, providing a holistic measure of obesity and fat distribution [14]. Compared to other indicators, WWI may be more sensitive in reflecting abdominal fat distribution because waist circumference directly correlates with visceral fat accumulation. Studies have shown that increased abdominal fat, especially visceral fat, is closely associated with various metabolic diseases. Visceral fat is a key driver of insulin resistance and a risk factor for diabetes mellitus. Excessive visceral fat accumulation leads to the excessive release of fatty acids, which disrupt insulin signaling and contribute to insulin resistance [15]. Moreover, individuals with high WWI values tend to exhibit higher levels of inflammation and oxidative stress [19,20]. Inflammatory mediators, such as TNF-α and IL-6, interfere with normal insulin function, ultimately leading to the onset of insulin resistance and an increased risk of developing prediabetes.

Wen et al. reported an association between NHHR and prediabetes risk, partly mediated by BMI. While our findings similarly highlight the predictive role of NHHR, we further demonstrate its mediating effect between WWI and prediabetes, offering new insight into how central obesity may contribute to prediabetes via lipid metabolism [21]. NHHR, as a comprehensive lipid metabolism index, reflects the risk of abnormal lipid metabolism and atherosclerosis [22]. An increase in NHHR is associated with a higher risk of developing type 2 diabetes and prediabetes, with its predictive efficacy surpassing that of traditional lipid metrics [11]. Other studies have also found that NHHR is a predictor of mortality in some chronic diseases [23]. Our findings align with these studies and further validate the potential of NHHR in prediabetes screening. Increased NHHR reflects an imbalance between non-HDL-C and HDL-C in the body, which plays a significant role in the onset of diabetes. Disturbances in lipid metabolism may also be a significant factor influencing the development of prediabetes. Additionally, the liver plays a central function in lipid metabolism, and hepatic fat accumulation and lipid metabolism disorders are closely related to the progression of diabetes and prediabetes. Elevated NHHR may indicate abnormalities in hepatic lipid metabolism, which further exacerbate insulin resistance and glucose metabolism disorders, thereby promoting the development of prediabetes [24].

Elevated WWI is associated with visceral fat accumulation, which is closely linked to insulin resistance and inflammatory responses. It also affects systemic lipid metabolism through the secretion of adipokines (e.g., free fatty acids, tumor necrosis factor-α) [4,25], which in turn influences NHHR. The buildup of abdominal fat promotes the release of adipokines, such as TNF-α and IL-6, activating signaling pathways associated with metabolic disorders [26]. Previous studies have shown that obese patients tend to exhibit higher levels of non-HDL-C [27].Non-HDL-C is a significant promoter of atherosclerosis, and its elevation is closely associated with the development of prediabetes and type 2 diabetes [28]. Recent evidence has challenged the notion that higher HDL-C is always protective [29]. Large-scale studies and meta-analyses have shown that extremely high HDL-C levels may paradoxically increase the risk of adverse cardiovascular outcomes. Additionally, non-HDL-C includes cholesterol carried by very low-density lipoproteins (VLDL-C), which also play a role in cardiometabolic risk [30,31]. Obesity is often accompanied by metabolic syndrome, including insulin resistance, hyperglycemia, and lipid metabolism disorders [32]. These metabolic abnormalities may lead to an increase in non-HDL cholesterol, affecting NHHR. Therefore, WWI may ultimately contribute to the elevation of NHHR and influence the development of prediabetes through several pathways, including promoting visceral fat accumulation, altering lipid metabolism, and increasing inflammatory responses.

The results of this study have important public health implications. The prevalence of prediabetes continues to rise globally, and many patients are unaware that they are at high risk at this stage. Therefore, early screening for prediabetes is crucial. Traditional screening methods for diabetes typically rely on fasting glucose and glucose tolerance tests, which can be cumbersome and require high levels of patient compliance. WWI and NHHR are calculated using physical measurements and lipid data, making them relatively simple and inexpensive to implement. Particularly in resource-limited areas, these measures offer a novel approach to prediabetes screening. By monitoring WWI and NHHR, high-risk individuals can be identified early, allowing for timely intervention to reduce the incidence of prediabetes and its complications. These results are consistent with previous evidence suggesting that anthropometric and lipid indices, including WWI and NHHR, play important roles in predicting disease risk [11,33].

Although this study provides valuable insights, several limitations remain. First, the data were derived from NHANES. Although NHANES data are representative, the cross-sectional design restricts the ability to establish causality. Therefore, future longitudinal studies may better validate the predictive value of WWI and NHHR in the development of prediabetes. Second, this study only analyzed data from U.S. adults, which may not fully reflect differences across age groups or ethnic populations.

## 5. Conclusion

This study explored the relationship between WWI, NHHR, and the risk of prediabetes. Both WWI and NHHR were significantly associated with the risk of prediabetes, and NHHR partially mediated the effect of WWI on prediabetes. This study provides a new theoretical basis for early prediabetes screening, prevention, and control. In the future, lifestyle interventions targeting WWI and NHHR may be effective in preventing prediabetes.

## Supporting information

S1 Fig Flow chart of the population included in this study. (JPG)
