# Correction: Role of weight-adjusted waist circumference index and non-high-density lipoprotein cholesterol/high-density lipoprotein cholesterol ratio in prediabetes risk: A mediation analysis

**DOI:** 10.1371/journal.pone.0345167

**Published:** 2026-03-16

**Authors:** The PLOS One Staff

There are errors in the author affiliations. The correct affiliations are as follows:

Wei Mi^1^, Cuixiao Wang^2^

**1** School of Nursing, Hunan University of Medicine, Huaihua, China, **2** Hunan University of Medicine General Hospital, Huaihua, China.

The publisher apologizes for the error.
