# Supplementary figures and images for: Role of weight-adjusted waist circumference index and non-high-density lipoprotein cholesterol/high-density lipoprotein cholesterol ratio in prediabetes risk: A mediation analysis
Source: PLoS One. 2025 Sep 25;20(9):e0331866. doi: 10.1371/journal.pone.0331866 (PMC12463217; doi:10.1371/journal.pone.0331866)

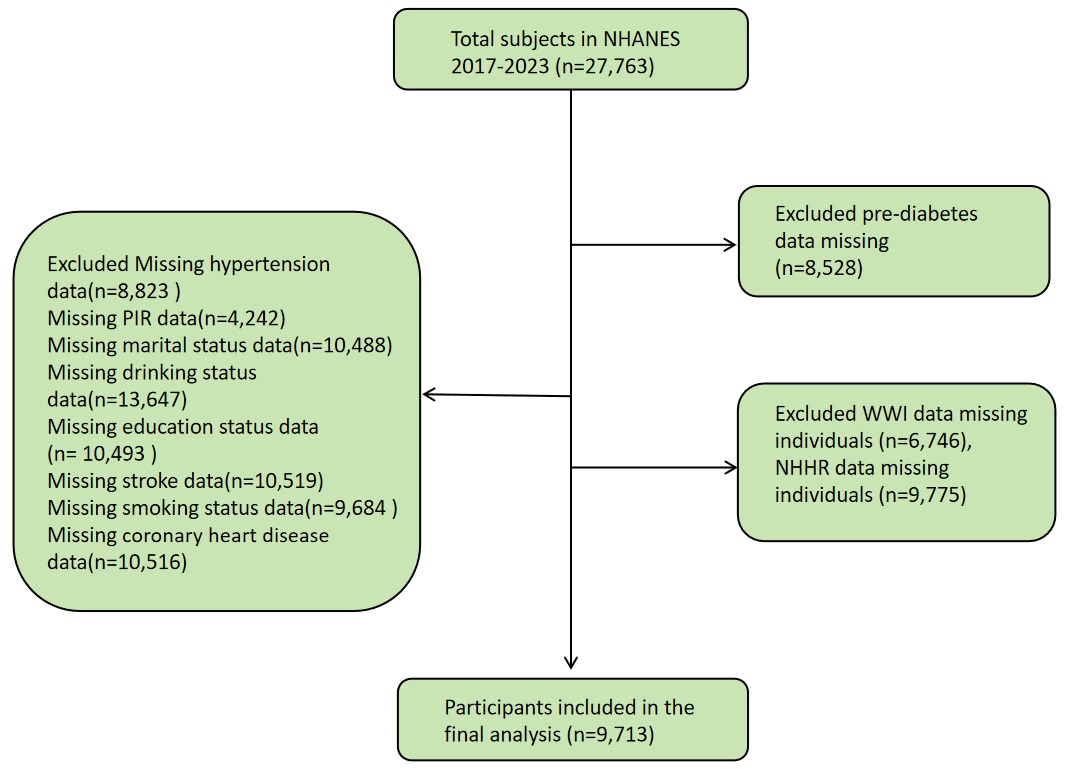

Supplement: S1 Fig — (JPG) [file pone.0331866.s001.jpg]
